# Total Hip Arthroplasty for Femoral Neck Fracture after Postoperative Intertrochanteric Fracture in a Patient with Spontaneous Fused Hip

**DOI:** 10.1155/2019/8654194

**Published:** 2019-12-12

**Authors:** Saori Niitsu, Shohei Okahisa, Yuki Fujihara, Yu Takeda, Shigeo Fukunishi

**Affiliations:** ^1^Department of Orthopedic Surgery, Hyogo College of Medicine, Japan; ^2^Nishinomiya Kaisei Hospital, Japan

## Abstract

A 64-year-old woman with a spontaneous fused hip sustained a left femoral neck fracture. It was revealed that her left hip joint had a long-standing spontaneous hip fusion due to end-stage osteoarthritis. Additionally, she sustained an ipsilateral femoral intertrochanteric fracture and underwent osteosynthesis using a dynamic hip screw 8 years ago. The one-stage THA was successfully treated with no major complications and good functional recovery was obtained. The hip range of motion improved remarkably at one year after surgery. The Modified Harris Hip Score improved from an estimated 70 points before fracture to 95 points at final follow-up.

## 1. Introduction

The conversion procedure to total hip arthroplasty (THA) for a patient with hip arthrodesis and spontaneous fused hip presented surgical difficulty compared to the common primary THA [1–3]. This challenging procedure often provides significant improvement to the patient's quality of life. However, higher complication rates have been reported [4–8]. On the other hand, a proximal femoral fracture in a patient with a fused hip rarely develops [9]. Previously, various successful procedures for osteosynthesis have been reported [10–20], and patients were able to acquire the same level of activities of daily living (ADL) as before the fracture. To the best of our knowledge, there have been no reports that describe one-stage THA for a patient with a proximal femoral fracture under a long-standing fused hip. In the present case report, a 64-year-old woman sustained a femoral neck fracture under a long-standing spontaneous hip fusion. Additionally, the patient had undergone osteosynthesis for an intertrochanteric fracture 8 years before this femoral neck fracture. We successfully treated this very rare fracture through one-stage THA.

## 2. Case Study

**Figure 1:**
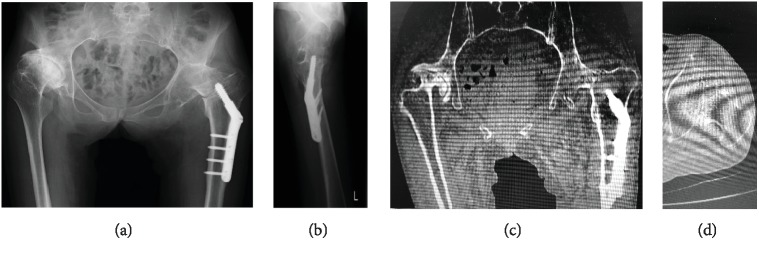
The plain radiograph and CT of both hip joints of a 64-year-old woman. Left hip joint showed the femoral neck fracture after postoperative intertrochanteric fracture in the fused hip. Right hip joint shows severe osteoarthritis due to DDH. (a) Anteroposterior view; (b) lateral view; (c) sagittal view of CT image; (d) axial view of CT image.

A 64-year-old woman who worked at a laundry and dry-cleaning store was admitted to our hospital due to a fall. She complained of left hip pain and was unable to walk. It was revealed that her left hip joint had a long-standing spontaneous hip fusion due to end-stage osteoarthritis with developmental hip dysplasia (DDH) for more than 30 years. Additionally, she sustained an ipsilateral femoral intertrochanteric fracture 8 years ago and underwent surgery with osteosynthesis using a dynamic hip screw. The physical findings at the initial visit showed spontaneous pain and tenderness around the left hip joint, and she was unable to move her left leg. The left hip joint was fixed at flexion 0° and abduction 0°, and no unusual rotation was detected in the neutral limb position. Plain radiograph of the left hip joint revealed a fused hip and a nondisplacement femoral neck fracture at the tip of the lag screw which was inserted for the intertrochanteric fracture 8 years ago (Figures 1(a) and 1(b)). Moderate deformity of the proximal femur was present with a femoral anteversion of 14° and a neck-shaft angle of 118°. In addition, severe osteoarthritis due to DDH was showing in the right hip joint. The leg length discrepancy was determined by measuring the distance from the anterior-superior iliac spine to the medial malleolus of the ankle. The actual leg length of the affected side (left limb) was 10 mm shorter than the other. Similar findings were found in CT examination (Figures 1(c) and 1(d)). From the above, it was diagnosed as a femoral neck fracture after postoperative intertrochanteric fracture in the fused hip.

It was estimated that the ADL of the patient before the fracture was that she could walk without a cane and no support was needed during her daily living; however, she had right hip pain with osteoarthritis.

**Figure 2:**
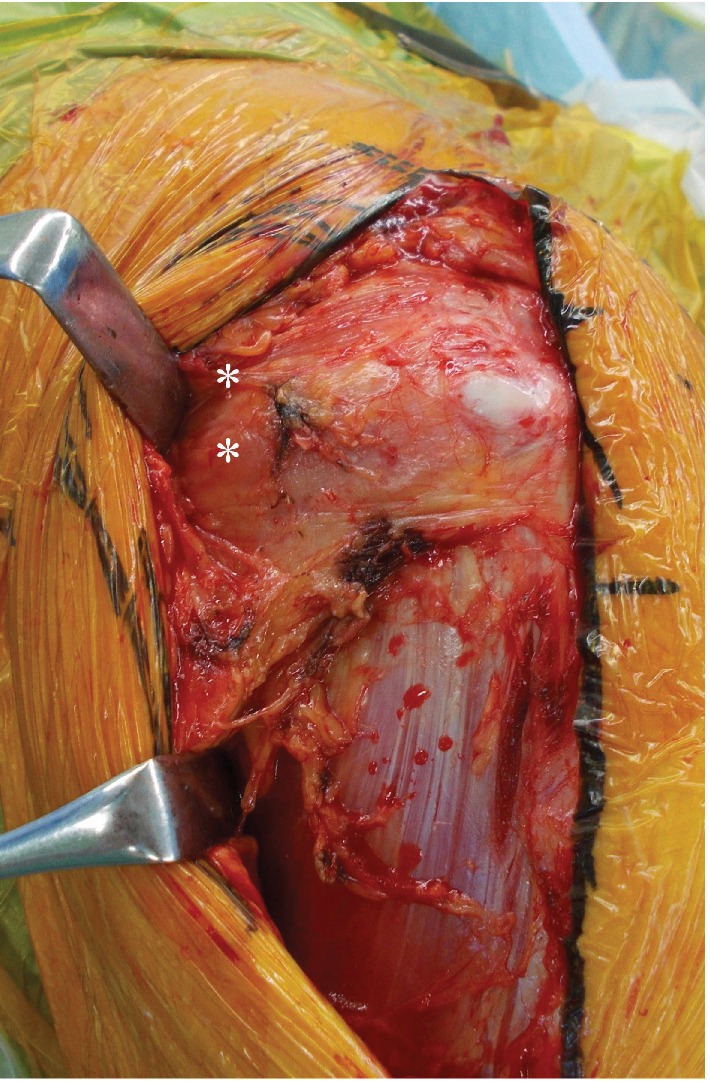
Intraoperative finding. White asterisks ∗ showed atrophy with fatty degeneration in gluteus medius.

**Figure 3:**
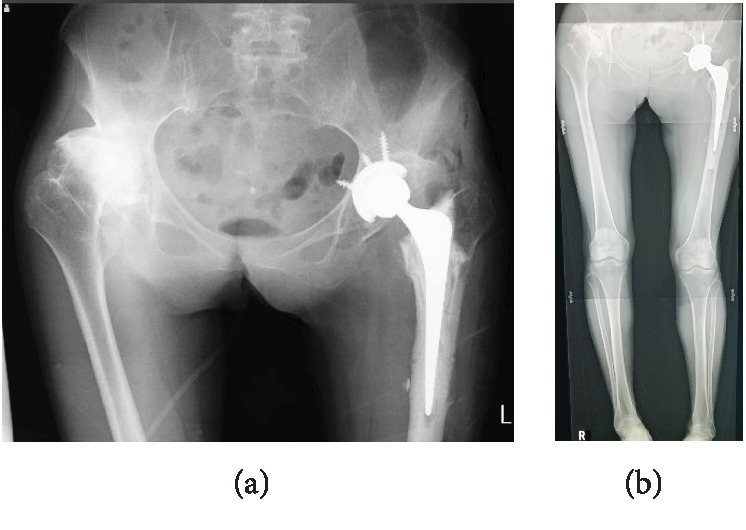
Postoperative plain radiograph. Hybrid THA was performed. (a) Anteroposterior view; (b) whole lower extremities in standing position.

After discussing treatment options, we selected one-stage THA and to extract the dynamic hip screw. In the preoperative planning, we generally proposed to place the cup at the original hip center; however, in this case, the cup could only be placed at 5 mm higher than the hip center in order to avoid the cup CE angle of less than 0 degrees. Surgery was performed at the lateral decubitus position without navigation under general anesthesia, and the modified Hardinge approach was used to take down hip fusion surgery after extracting the dynamic hip screw. During surgery, atrophy with fatty degeneration in the gluteus medius was observed (Figure 2). A neck cut was performed through the fracture line. Subsequently, iliopsoas and adductor tenotomies were performed before the preparation of the acetabulum and femur. Before the acetabulum preparation, we confirmed the original acetabulum, the height of the tear drop line, and the inclination angle for acetabular reaming by intraoperative fluoroscopy. We performed the reaming of the acetabulum along with the fused femoral head. Finally, we reconfirmed the depth of the reamer to avoid the perforation into the medial wall of the acetabulum by fluoroscopy while final reaming was performed. Subsequently, anterior and posterior excessive bone around the cup which originated from the femoral head and osteophyte was removed. After the preparation of the acetabulum and rasping of the femur, a cementless cup (Trident Acetabular Shell, Stryker Orthopedics, NJ, USA), a cemented stem (Exeter V40 Femoral Stem, Stryker Orthopedics, NJ, USA), a ceramic 32 mm head (BIOLOX Delta V40 Ceramic Head, Stryker Orthopedics, NJ, USA), and a nonelevated ultrahigh molecular weight polyethylene liner (Trident X3 Insert, Stryker Orthopedics, NJ, USA) were implanted. An impingement test was performed after implantation and neither bony impingement nor implant-bone impingement were confirmed. A postoperative rehabilitation program was instilled to allow free mobilization and full weight-bearing exercise one day after surgery. The patient was able to walk with a walker two weeks after surgery and was discharged with a T-cane one month after surgery. One year after surgery, the patient was able to walk without a cane, and the hip range of motion improved remarkably with flexion 100°, extension 10°, abduction 30°, internal rotation 30°, and external rotation 40°. Postoperative radiograph with the whole lower extremities in standing position showed the subjective leg length to be 4 mm longer in the left limb. We are considering future THA for the right hip due to severe osteoarthritis (Figures 3(a) and 3(b)). The Modified Harris Hip Score improved from an estimated 70 points before fracture to 95 points at the final follow-up. The patient has returned to her previous work.

## 3. Discussion

There are a few available reports regarding proximal femoral fractures in arthrodesis or spontaneous fused hip joints [10–20]. Sponseler et al. reported in 1984 that the rate of proximal femoral fracture in this condition was 73% (2/53 cases) [9]. Therefore, the appropriate treatment guideline has not been established. This rare fracture could cause different mechanical stresses at the fracture site compared to common proximal femoral fractures. The fractured fragment was divided into two fragments, which consisted of the pelvis with proximal femur as the proximal fragments and the distal femur with the long lever arm of the lower extremity as the distal fragment. The large rotational stress and the shear stress were produced at the fracture site until bony fusion was performed [11, 20]. Therefore, it was difficult to maintain sufficient stability with conservative treatment to achieve bony fusion; thus, there have been no reports that recommend conservative treatment. There are two surgical options that may be considered: open reduction and internal fixation (ORIF) and THA. ORIF cannot be expected to improve the leg length discrepancy and acquisition of a hip range of motion; however, if the bony fusion is obtained, the patient may be able to acquire the same ADL from before the fracture. Most of the previous reports on treatments with ORIF were related to intertrochanteric fracture or subtrochanteric fracture, and successful results with various methods and implants for osteosynthesis have been reported [12, 15, 16, 19, 20]. However, it is necessary to maintain a very rigid fixation on the fracture site. Asakawa et al. and Manzotti et al. have reported that double plate fixation is needed for rigid fixation [12, 20]. Pascarella et al. have reported a case of recurrent subtrochanteric nonunion due to inadequate fixation [21]. On the other hand, conversion of a fused hip to THA can restore function and enhance patients' quality of life (QOL) [1–3]. The conversion of a fused hip to THA could obtain an improvement in the hip range of motion, leg length discrepancy, and adjacent joint disorder. Therefore, patients seek conversion to THA, hoping to alleviate symptoms. However, a systematic review by Jauregui et al. described that specific postoperative complications were 5.3% for infection, 4.7% for nerve-related complications, 2.6% for instability, 6.2% for loosening, 13.1% for abductor-related complications, and 1.2% for venous thrombotic events [22]. Another study by Richards and Duncan reported significantly worse clinical outcomes and patient satisfaction as well as higher complication rates compared to common primary and revision THA [23]. Regardless of the higher complication rate, patient satisfaction and postoperative outcomes were generally good [6, 8, 22]. Regarding surgical techniques, it was difficult to secure adequate visualization of the surgical field due to the contracture of the soft tissue and a lack of hip movement [7, 21]. Additionally, the level of the neck cut and the original acetabulum were difficult to identify due to the deformity of the pelvis and fused proximal femur [6–8]. Malpositioning of the femur, which included a high femoral neck-shaft angle, unusual anteversion, and flexion-abduction contracture, made the preparation of the femur difficult [6, 8, 12]. Furthermore, poor visualization, insufficient bone stock, and loss of the surgical landmark made it difficult to set the acetabular cup at the original acetabulum.

In the present case, we had several technical advantages on the surgery. First, fortunately, no abnormal contracture was present, and the hip joint had been fused in the neutral limb position. Second, the fracture line in the femoral neck was nearly consistent with the required neck cut line of THA. The femur could be moved a little at the site of the fracture; therefore, we were able to obtain a sufficient surgical field. For that reason, our approach for the surgery did not need trochanteric osteotomy, although Morsi and Richards and Duncan recommend the lateral transtrochanteric approach with trochanteric osteotomy for sufficient visualization of the surgical field [6, 23]. Third, only a moderate deformity of the proximal femur was present with a femoral anteversion of 14° and a neck-shaft angle of 118°. Additionally, a major leg length discrepancy was not present in this case, which enabled us to perform femoral stem preparation as usual.

If severe proximal femoral deformity was present, additional osteotomy in the proximal femur might have been needed. Additionally, if abnormal femoral anteversion was present, version control by modular stem or cemented stem would have been needed to avoid postoperative dislocation. For the acetabulum preparation, fluoroscopy was used for acetabular reaming to confirm the position of the original acetabulum. We could not use navigation in this case; however, CT-based navigation could be safer and more accurate. Postoperative outcomes were satisfactory at final follow-up at one year after surgery. There were no major complications, such as dislocation, deep venous thrombosis, or deep infection encountered during the study period.

The limitations associated with this case report include the fact that the postoperative follow-up period was quite short, and that future observation of progress is necessary. However, to the best of the authors' knowledge, this is the first report with one-stage THA for a femoral neck fracture after postoperative intertrochanteric fracture in a fused hip.

## 4. Conclusion

One-stage THA was successfully treated and good functional recovery was obtained in a patient with a femoral neck fracture after a postoperative intertrochanteric fracture in a spontaneous fused hip.
